# The efficacies and biomarker investigations of antiangiogenic agents and PD-1 inhibitors for metastatic soft tissue sarcoma: A multicenter retrospective study

**DOI:** 10.3389/fonc.2023.1124517

**Published:** 2023-02-22

**Authors:** Zhiyong Liu, Xin Wang, Jiaqiang Wang, Peng Zhang, Chao Li, Bangmin Wang, Songtao Gao, Oufei Liu, Weitao Yao

**Affiliations:** ^1^ Department of Orthopedics, The Affiliated Cancer Hospital of Zhengzhou University and Henan Cancer Hospital, Zhengzhou, Henan, China; ^2^ Department of Orthopedics, Henan Provincial People’s Hospital, Zhengzhou, Henan, China; ^3^ Department of Oncology, The Fifth Affiliated Hospital of Zhengzhou University, Zhengzhou, Henan, China

**Keywords:** angiogenesis, PD-1 inhibitor, soft tissue sarcoma, survival, prognostic biomarker

## Abstract

**Objective:**

To investigate the efficacy and safety of antiangiogenesis-immunotherapy in patients with advanced STS in China, and to explore the potential factors of prognosis.

**Patients and Methods:**

This retrospective study was conducted at three hospitals in China, and the patients with metastatic STS who were ineligible for or declined anthracycline-based chemotherapy received antiangiogenic agents (anlotinib or apatinib) plus programmed death-1 (PD‐1) inhibitors (camrelizumab or sintilimab) between June 2019 and May 2022. The primary endpoint was progression-free survival rate at 6 months (6-month PFSR), and the secondary endpoints were objective response rate (ORR), disease control rate (DCR), progression-free survival (PFS), and overall survival (OS) and toxicity. Biomarkers that might affect the prognosis were explored.

**Results:**

Thirty-nine patients were included: five patients with alveolar soft tissue sarcoma (ASPS) and 34 with non-ASPS. With a median follow-up of 18.2 months, the 6-month PFSR was 51.3%, with the ORR of 20.5% and DCR of 76.9%. The median PFS and OS were 7.0 months and 17.2 months. The 6-month PFSR for patients with ASPS and non-ASPS was 80.0% and 47.1%, respectively. The most common adverse events were hypothyroidism (56.4%), followed by fatigue (46.2%), and hypertriglyceridemia (43.6%). No treatment-related deaths were observed. Patients with low baseline NLR (NLR < 4) had better 6-month PFSR than those with high NLR (NLR ≥ 4) (82.4% vs. 31.6%).

**Conclusion:**

Antiangiogenic agents plus PD-1 inhibitors showed acceptable toxicity and promising efficacy in patients with advanced STS, especially patients with ASPS, and a low NLR might serve as a reliable biomarker for 6-month PFSR, PFS, and OS. It provides a reference for randomized controlled trials.

## Introduction

Soft tissue sarcoma (STS), a rare but heterogeneous group of malignant tumors originating from mesenchymal tissue, is characterized by 1% of all adult malignancies and more than 50 histopathologies (1, 2). Nearly 40,000 new cases are diagnosed each year in China. Although surgery and/or radiation therapy is considered the standard treatment for most localized STS, more than 30% of patients with high-risk STS would suffer tumor recurrence and metastasis after aggressive treatment (3). For decades, palliative chemotherapy based on doxorubicin has become one of the most effective therapies for advanced disease, which provides an objective response rate (ORR) of 5%-20%, a median progression-free survival (PFS) of 4.2 months and a median overall survival (OS) of 14 months (3). There is no consensual treatment after failure or intolerance of doxorubicin, second and further-line cytotoxic agents approved as salvage treatment only showed some signs of activity with poor tolerance including gemcitabine plus docetaxel, ifosfamide, and dacarbazine (3). Therefore, new drugs and combination strategies are urgently needed to improve survival.

Preclinical studies have demonstrated that angiogenesis plays an important role in multiple pathological conditions including tumor growth, progression, and metastasis (4). The vascular endothelial growth factor (VEGF)/VEGFR2 receptors (VEGFRs) pathway is considered to be one of the most important signaling pathways in STS (5). Clinical trials have confirmed that several antiangiogenic agents could significantly prolong PFS and/or improve quality of life including pazopanib, cabozantinib, and regorafenib (6). These agents are widely used in second-line therapy due to their efficacy in terms of PFS and favorable adverse effects (AEs). Anlotinib and apatinib, recognized as active drugs for advanced STS, have become the two most widely used drugs in China since June 2019 because of their low price and good accessibility (5, 7). Preclinical studies suggest that the two drugs exhibit strong anti-angiogenesis and inhibition of tumor growth and progression in malignancies. Anlotinib is a broad-spectrum tyrosine kinase inhibitor that can effectively block multiple protein kinases and their interactions, including VEGFR, PDGFR, and FGFR, with the IC50 of VEGFR2 of 2nM (7). Meanwhile, apatinib could highly and selectively bind to VEGFR-2 with the IC50 of 1nM (5). Additionally, the two agents could induce changes in the tumor microenvironment by reducing the levels of tumor-associated macrophages to enhance antitumor activity (8). Prospective studies such as ALTER0203 showed that both anlotinib monotherapy and apatinib monotherapy had moderate antitumor efficacy in advanced STS, with an ORR of 10.13%-13% and a median PFS of 3.35-6.27 months (4, 9).

Immune checkpoint inhibitors have substantially improved the treatment in a wide range of cancers and have been explored as a novel strategy for patients with advanced STS. Preclinical studies have confirmed that PD-L1 expression varied in STS ranging from 0 to 100% and in its independent negative prognostic value in poor outcomes (10, 11). These suggest that the PD-1/PD-L1 axis may be a potential target for immunotherapy. PD-1 inhibitors can bind to the PD-1 receptor and block the connection between the PD-1 receptor and PD-L1 and PD-L2. Increasing studies have shown that several subtypes could benefit from PD-1 inhibitor-based therapy, especially alveolar soft tissue sarcomas (ASPS), undifferentiated pleomorphic sarcomas (UPS), leiomyosarcoma (LMS), and dedifferentiated liposarcoma (DDLPS) (12). The SARC028 study and its expansion showed that 10%-40% tumor response was observed in patients with advanced UPS and DDLPS who were administered with pembrolizumab (13, 14). With similar pharmacological mechanisms to pembrolizumab, camrelizumab (Jiangsu Hengrui Pharmaceuticals Co) and sintilimab (Innovent Biologics) are two novel PD-1 inhibitors developed in China. Based on clinical trials, they have been approved for the treatment of multiple tumors including lymphoma, renal cell carcinoma, and hepatocellular carcinoma. There is increasing evidence preliminarily showing the anti-cancer effect of the therapy based on camrelizumab or sintilimab in advanced STS (15, 16). However, due to the cold tumor microenvironment with two mutations per DNA megabase (17), 23% ± 13% of CD8+ lymphocytes (18), 6.6% of programmed death-ligand 1 (PD-L1) (19), PD-1 inhibitors monotherapy failed to show satisfactory tumor response and durable antitumor control in most STS. Therefore, the combination therapy of PD-1 inhibitors plus other agents that would overcome the immune barrier and potentially improve prognosis is becoming an option (20).

Antiangiogenic agents and PD-1 inhibitors attracted significant attention for their synergistic anti-tumor effect and encouraging efficacy in some malignancies including hepatocellular carcinoma (HCC) (21), renal cell carcinoma (22), and melanoma (23). Some patients received an off-label combination treatment of antiangiogenic agents (apatinib or anlotinib) and PD-1 inhibitors (camrelizumab or sintilimab) in China. This study aimed to retrospectively evaluate the safety and efficacy of such a combination therapy and to provide a reliable reference for prospective studies in advanced STS. Additionally, we also preliminarily integrated clinical characteristics to identify the potential biomarkers for response and survival.

## Patients and methods

### Study design and patients

We retrospectively analyzed patients with metastatic STS treated with antiangiogenic agents plus PD-1 inhibitors in Henan Cancer Hospital, the Fifth Affiliated Hospital of Zhengzhou University, and Henan Provincial People’s Hospital between June 2019 and May 2022. This study complied with the principles of Helsinki, met the requirements of the ethics committee and was approved by the ethics committees of each institute. All participants provided written informed consent before treatment.

Patients were included according to the main criteria: 1. Age 18 to 70; 2. The performance status of Eastern Tumor Cooperative Group (ECOG) is 0-2; 3. The pathological diagnosis included ASPS, UPS, synovium, LMS, epithelioid sarcoma (ES), fibrosarcoma, etc. 4. At least one measure based on the Response Evaluation Criteria for Solid Tumors (RECIST) 1.1; 5. Complete medical history and follow-up records; 6. Not eligible for or refusing first-line chemotherapy; and 7. Progressive disease within 6 months before combination treatment. Patients were excluded if they presented contraindications of antiangiogenic agents and/or PD-1 inhibitors including coagulation dysfunction, active asthma, etc.

### Treatment

The combination strategy was developed according to previous treatment, individual characteristics, patient willingness, and economic considerations. All patients received at least one cycle of combination therapy with antiangiogenic agents and PD-1 inhibitors. The antiangiogenic agents are apatinib and anlotinib, and the PD‐1 inhibitors are camrelizumab and sintilimab. Anlotinib (12 mg/day or 10 mg/day) was taken orally from day one to 14 every 21 days and apatinib (500 mg/day or 250 mg/day) was taken orally daily. Simultaneously, patients were intravenously administered with sintilimab 200 mg or camrelizumab 200 mg every three weeks, The combination therapy was repeated every three weeks until PD, intolerance to toxicity, or refusal of treatment by patients or physicians. Patients experienced dose delay, dose reduction, treatment interruption, and discontinuation of antiangiogenic drugs based on the grade of toxicity. However, the dose of PD-1 inhibitors was not allowed to be adjusted. If one of two regimens could not be tolerated, the other could be continued.

### Assessment

Radiological data were collected based on CT scan and/or MRI at baseline and every 2-4 cycles thereafter until toxicity intolerance or progressive disease (PD). Clinical characteristics and hematological data were collected at baseline. Tumor assessments were performed and divided into complete response (CR), partial response (PR), stable disease (SD), and PD based on RECIST 1.1. The key endpoint was the progression-free survival rate at six months (6-month PFSR), with PFS defined as the interval between the start of treatment and PD or death. Secondary endpoints included ORR, disease control rate (DCR), PFS, OS, and adverse events (AEs). The ORR was calculated as the percentages of CR and PR during the treatment, and DCR was determined as the percentages of CR, PR, and SD. OS was defined as the interval from the start of treatment to death. All AEs were collected and assessed based on the Common Terminology Standard for Adverse Events (CTCAE) 5.0 criteria.

### Statistical analysis

Continuous variables are represented as mean ± SD or median (interquartile range), and categorical variables are represented as case (percentage). Pearson’s χ^2^ test or Fisher’s exact test were applied to analyze categorical variables in clinical characteristics. PFS and OS were generated by the Kaplan-Meier method with their 95% confidence interval (CIs) calculated using the Brookmeyer–Crowley method. The relationship between prognostic biomarkers and survival (PFS and OS) was evaluated by Cox proportional hazards regression models. A two‐sides *p* value less than 0.05 was considered statistically significant. Statistical analyses were performed using GraphPad Prism version 8.0 (GraphPad Software, La Jolla, CA) and IBM SPSS version 23.0 (IBM, Chicago, IL).

## Results

### Baseline characteristics

As detailed in **Table 1**, from June 2019 to May 2022, thirty-nine patients (19 men and 20 women) with metastatic STS were included with a median age of 45 (18-70) years. There were eight patients with synovial sarcoma, six with LMS and five with UPS, five patients with ASPS, and 15 with other subtypes. All patients had metastatic diseases. Lung was the most common metastatic lesion (59%), followed by lymph nodes (20.5%), liver (15.4%), and peritoneum (12.8%). Previous therapies were diverse: 32 (82.1%), 15 (38.5%), and 35 (89.7%) patients received prior surgery, radiotherapy, and chemotherapy, respectively. Twenty-two (56.4%) and 13 (35.8%) patients have previously received first-line and later-line chemotherapies, respectively. Five (12.8%) and three (7.7%) patients have received antiangiogenic agents and PD-1 inhibitors, respectively. The majority of sarcomas originated from the extremities and trunk, followed by the internal organs, head and face.

**Table 1:** Baseline characteristics and Treatment.

Characteristics	*N*=39
Age (years), median (range)	45(18-70)
Sex, n (%)	
Male	19(48.7%)
Female	20(51.3%)
ECOG	
0	25(64.1%)
1	8(20.5%)
2	6(15.4%)
Histology, n (%)	
Synovial sarcoma	8(20.5%)
Leiomyosarcoma	6(15.4%)
Alveolar soft part sarcoma	5(12.8%)
Undifferentiated pleomorphic sarcoma	5(12.8%)
Others*	15(38.5%)
No. of previous chemotherapies	
0	4(10.3%)
1	22(56.4%)
2	7(17.9%)
3	6(17.9%)
Previous tyrosine-kinase inhibitor	
Yes	5(12.8%)
No	34(87.2%)
Previous PD-1inhibitor	
Yes	3(7.7%)
No	36(92.3%)
Radiotherapy	
Yes	15(38.5%)
No	24(61.5%)
Metastatic sites	
Lung	23(59%)
Lymph nodes	8(20.5%)
Liver	6(15.4%)
Peritoneum	5(12.8%)
Others#	5(12.8%)
Primary lesion	
Extremity	22 (56.4%)
Trunk	6 (15.4%)
Other	11(28.2%)
Previous surgery	
Yes	32(82.1%)
No	7(17.9%)
Combination therapy	
An plus Sin	20 (51.3%)
Ap plus Car	10 (25.6%)
Ap plus Sin	5 (12.8%)
An plus Cam	4 (10.3%)

Others*, fibrosarcoma, clear cell sarcoma, epithelioid sarcoma, angiosarcoma, extraskeletal myxoid chondrosarcoma; Others #, bone, brain, cutaneous, and pleural; An, anlotinib; Sin, sintilimab; Ap, apatinib; Cam, camrelizumab.

### Treatment

Patients in this study received four combination therapies of antiangiogenic drugs and PD‐1 inhibitors, with the most common treatment being anlotinib plus sintilimab (n=20, 51.3%), followed by apatinib plus camrelizumab (n=10, 25.6%), apatinib plus sintilimab (n=5, 12.8%), and apatinib plus camrelizumab (n=4, 10.3%). Eighteen (46.2%), 10 (25.6%), six (15%), and five (12.8%) patients received anlotinib 12mg, apatinib 500mg, anlotinib 10mg, and apatinib 250mg as initial doses, respectively. The data cut-off was November 15, 2022, and six patients still continued treatment and 33 stopped treatment. Of the 33 patients, 29 discontinued treatment due to PD, three due to AEs, and one following the patient decision. The AEs that resulted in halting treatment included grade 3 pneumothorax (n=2), and grade 2 renal hemorrhage (n=1). Four hundred and thirty-seven 3-week cycles were given, with a median of 8.3 (1–39) and 11.5 (2–45) cycles of antiangiogenic drugs and PD‐1 inhibitors per patient, respectively. Twenty-four of the 39 patients died due to PD.

### Efficacy

**Figure 1:**
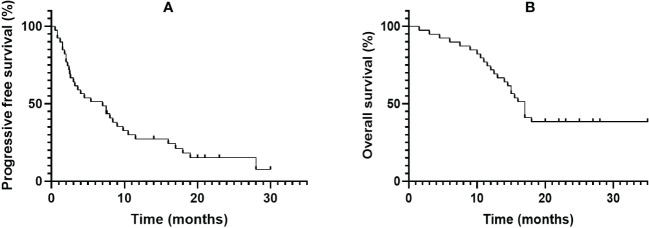
Kaplan–Meier estimates for progression-free survival **(A)** and overall survival **(B)**.

**Figure 2:**
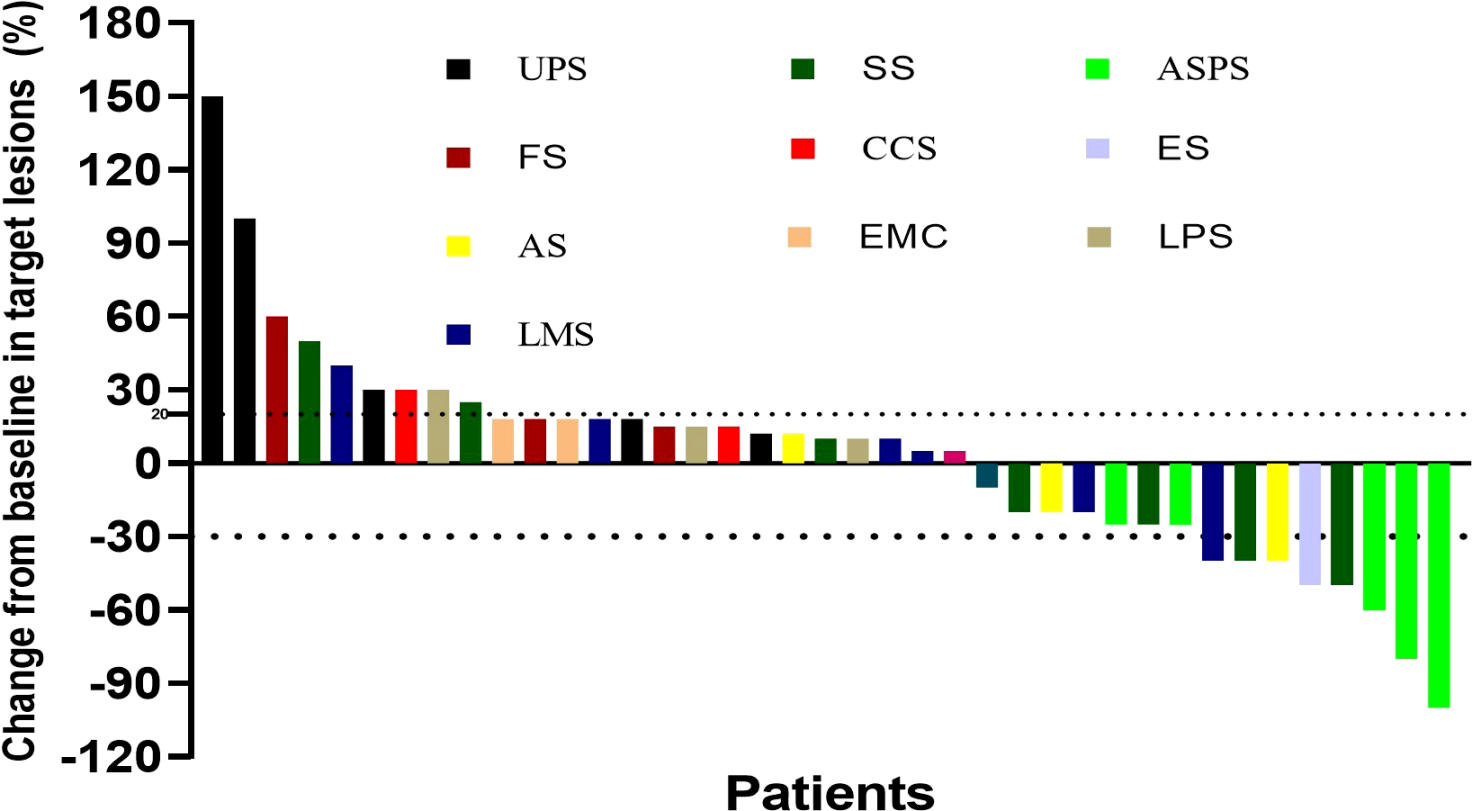
Best percentage change for the sum of tumor diameters from baseline. UPS, undifferentiated pleomorphic sarcoma; SS, synovial sarcoma; ASPS, alveolar soft tissue sarcoma; FS, fibrosarcoma; CCS, clear cell sarcoma; ES, epithelioid sarcoma; AS, angiosarcoma; EMC, extraskeletal myxoid chondrosarcoma; LPS, liposarcoma; LMS, leiomyosarcoma.

**Figure 3:**
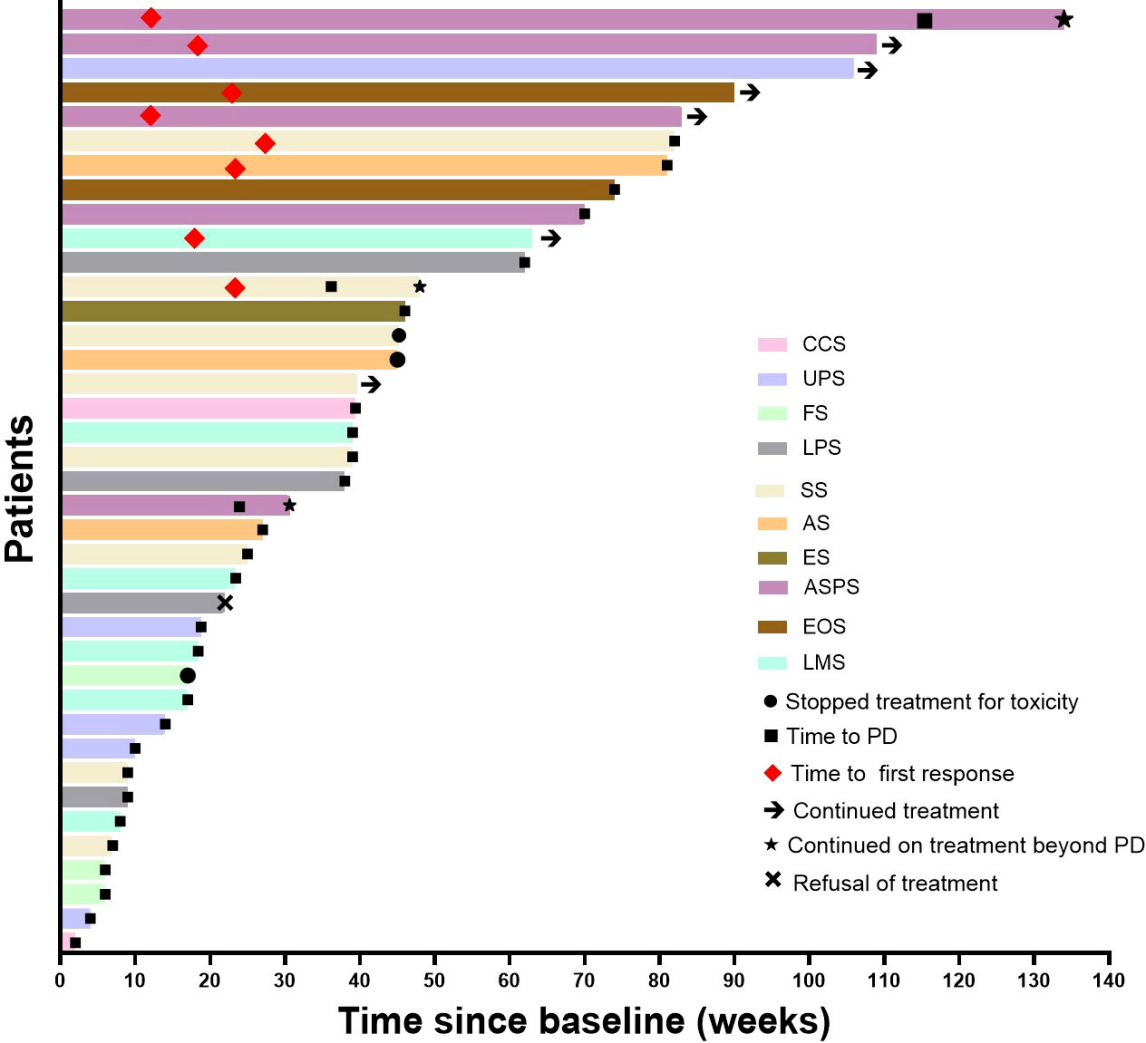
Time to response and duration of treatment. UPS, undifferentiated pleomorphic sarcoma; SS, synovial sarcoma; ASPS, alveolar soft tissue sarcoma; FS, fibrosarcoma; CCS, clear cell sarcoma; ES, epithelioid sarcoma; AS, angiosarcoma; EMC, extraskeletal myxoid.

Median follow-up was 18.2 (9.3–24.2) months, as shown in **Table 2**, the 6-month PFSR for all patients was 51.3%, and the median PFS was 7.0 months (95%CI, 2.2-11.5m) (**Figure 1**). The 3-month PFSR and 9-month PFSR were 48% and 48%, respectively. Tumor assessment at any point during treatment showed 1 CR, 7 PR, 22 SD, and 9 PD, yielding an ORR of 20.5% and a DCR of 76.9% (**Figure 2** and **Table 2**). The clinical benefit rate was 51.3%. Complete response occurred in one patient with ASPS, and partial response was observed in seven patients: two patients with ASPS, two with synovial sarcoma, and each one with angiosarcoma, with ES and LMS. The time to response and duration of treatment are shown in **Figure 3**. Of 21 patients with SD, six (28.6%) had target lesion reduction. The m-OS was 17.2 months (95%CI, 14.1-23.0 m), and the 12-month OS and 18-month OS were 71.8% and 41.0%, respectively.

**Table 2:** Best objective response.

	Total	ASPS	Non-ASPS	SS	LMS	UPS	Others
N	39	5	34	8	6	5	15
PFS at 6 months	51.3%	80%	47.1%	62.5%	33.3%	20%	53.3%
Median PFS (mo) (95% CI)	7.0 (2.2-11.5)	19.3 (5.4-26.1)	4.3 (2.4-8.4)	7.8 (1.5-18.1)	3.1 (1.5-14.6)	2.0 (0.8-20.7)	7.0 (2.2-11.5)
CR	1	1	0				
PR	7	2	5	2	1		2
SD	22	2	20	4	4	2	11
PD	9	0	9	2	1	3	3
ORR	20.5%	60%	14.7%	25%	16.7%	0	13.3%
DCR	76.9%	80%	73.5%	75%	80%	40%	86.7%
ORR at 6 months	17.9%	80%	11.8%	12.5%	16.7%	0	13.3%

ASPS, alveolar soft part sarcoma; SS, synovial sarcoma, LMS, leiomyosarcoma, UPS, undifferentiated pleomorphic sarcoma; PFS, progression-free survival; CR, complete response; PR, partial response; SD, stable disease; PD, progressive disease; ORR, objective response rate; DCR, disease control rate; Others, fibrosarcoma, clear cell sarcoma, epithelioid sarcoma, angiosarcoma, extraskeletal myxoid chondrosarcoma.

Efficacy was further assessed based on histopathologic subtypes (ASPS and non-ASPS). In five patients with ASPS, one patient had CR, two obtained PR, and two achieved SD. The ORR and DCR was 80% and 100%, respectively. The 6-month PFSR was 80%, the median PFS was 19.3 months (95%CI, 5.4-26.1m), and the median OS was not reached. Rapid and long-term responses were observed, the median time to response and the median duration of response were 3.2 months and 28.0 months. Among 34 patients without ASPS, none had CR, five obtained PR, and 20 achieved SD. The ORR and DCR were 14.7% and 73.5%, the 6-month PFSR was 47.1%, and the median PFS and OS were 4.3 months (95%CI, 2.4-8.4 m) and 15.0 months (95%CI, 11.5-20.0 m), respectively. We also analyzed the efficacy in common subtypes: Seven patients with synovial sarcoma (6-month PFSR, 62.5%; median PFS, 7.8 months (1.5-18.1m); median OS, 17.5 months (95%CI, 1.5-18.1m)), five patients with UPS (6-month PFSR, 20%; median PFS, 3.0 months (0.8-20.7m); median OS, 10.5 months (95%CI 0.5 m-NA)), and six patients with LMS (6-month PFSR, 33.3%; median PFS, 3.1 months (1.5-14.6 m); median OS,13.5 months (95%CI 5.4 m-NA)). The median OS has a tendency to be extended.

### Safety

The toxicity profile of the combination treatment was in line with what have been reported in previous studies (**Table 3**). Approximately 64.1% of patients (25/39) experienced at least one adverse event of any grade. The most common AEs were hypothyroidism (56.4%), fatigue (46.2%), and hypertriglyceridemia (43.6%). Grade 3 and 4 AEs occurred in 4 patients (10.3%) including pneumothorax (n=2, 5.1%), cytokine release syndrome (n=1, 2.6%), and hypertension (n=1, 2.6%). Six patients experienced dose modification or discontinuation due to antiangiogenic agents, while only two patients experienced suspension or permanent discontinuation caused by PD-1 inhibitors. No patients died from AEs related to the combination treatment.

**Table 3:** Adverse events.

Adverse events	Any grade, n (%)	Grade 1-2, n (%)	Grade 3 or 4, n (%)
Hypothyroidism	22 (56.4%)	22 (56.4%)	0
Fatigue	18 (46.2%)	18 (46.2%)	0
Hypertriglyceridemia	17 (43.6%)	17 (43.6%)	0
Nausea or vomiting	16 (41.0%)	16 (41.0%)	0
Elevated alanine aminotransferase	16 (41.0%)	16 (41.0%)	0
Elevated gamma glutamyltransferase	15 (38.5%)	15 (38.5%)	0
Hypercholesterolemia	15 (38.5%)	15 (38.5%)	0
Hand-foot syndrome	12 (30.8%)	12 (30.8%)	0
Hypertension	7 (17.9%)	6 (15.4%)	1 (2.6%)
Proteinuria	7 (17.9%)	7 (17.9%)	0
Arthralgia or myalgia	5 (12.8%)	5 (12.8%)	0
Cough	4 (10.3%)	4 (10.3%)	0
Pneumothorax	4 (10.3%)	4 (10.3%)	2 (5.1%)
Capillary hemangiomas	2 (5.1%)	2 (5.1%)	0
Anorexia	2 (5.1%)	2 (5.1%)	0
Cytokine release syndrome	1 (2.6%)	1 (2.6%)	1 (2.6%)
Renal hemorrhage	1 (2.6%)	1 (2.6%)	0

Three treatment-related serious AEs were reported. Among these, two cases were attributed to antiangiogenic agents (malignant hypertension and pneumothorax), while the other one was cytokine release syndrome in one patient with SS treated with apatinib combined with camrelizumab which is rarely reported.

### Potential factors of treatment response and prognosis

We then firstly evaluated the relationship between prognosis (PFS ≥ 6 months and ORR) and several easily available factors including histopathology, baseline NLR, baseline LMR, and baseline absolute lymphocyte count. A total of 36 patients were included because three patients had infections, antibiotic treatment, and other factors. We found that a low baseline NLR was associated with tumor response (CR and PR) (*p* = 0.041, **Figure S1**) and a high rate of PFS at six months (*p* = 0.002, **Figure S1**). A high baseline LMR was associated with tumor response (CR and PR) (*p* = 0.036, **Figure S1**). ASPS tended to have a better tumor response (CR and PR) and a high rate of PFS at six months, but they were not statistically significant. Neither baseline LMR nor baseline absolute lymphocyte count was related to PFS ≥ 6 months or tumor response. Subsequently, we performed the association between survival (PFS and OS) and clinical characteristics including age, sex, ECOG, etc. Of these factors, Cox analysis identified the independent predictors for PFS as NLR and absolute lymphocyte count at baseline, while the independent predictor for OS was NLR at baseline. In detail, patients with low baseline NLR obtained significantly longer PFS than those with high baseline NLR {*p* = 0.008, HR=4.278, 95%CI (1.469-12.463)], and patients with absolute normal lymphocyte count had greater PFS than those with lymphocytopenia [*p* = 0.026, HR=0.361, 95%CI (0.147-0.885)]. Patients with low baseline NLR obtained significantly longer OS than those with high NLR [*p* = 0.009, HR=4.788, 95%CI (1.480-15.485)] (**Table S1**).

## Discussion

Antiangiogenic agents plus PD‐1 inhibitors show synergistic antitumor effects and have been considered an efficacious strategy for several cancers. However, only limited studies have been reported on this strategy in advanced STS. Herein, our study preliminarily explored the safety and efficacy of antiangiogenic agents plus PD-1 inhibitors in patients with metastatic STS. The PFSR at six months was 51.3%, which significantly exceeds the cut-off value for the activity of 15% recommended by the European organization for research and treatment of cancer (EORTC) (24). The ORR was 20.5%, with a median PFS of 7.0 months and a median OS of 17.2 months, respectively. The efficacy differs between pathological subtypes. The PFSR at six months in ASPS and non-ASPS were 80% and 47.1%, respectively, and the median PFS in ASPS and non-ASPS were 19.3 months and 4.3 months, respectively. This promising efficacy in this study provides a reference for similar clinical studies in the future.

Our study showed that antiangiogenic agents plus PD-1 inhibitors were as effective or better than any single agent monotherapy in advanced STS compared to historical data. The ALTER 0203 study suggested that anlotinib showed an ORR of 10.13%, and a median PFS of 6.27 months in 158 patients with advanced STS (9). Prospective studies showed that apatinib monotherapy showed modest efficacy with ORR of 9%, PFS of 7.87 months, and 12-week PFS of 70% (25). Prospective data on sintilimab monotherapy and camrelizumab monotherapy in STS were lacking. Thus, we referred to the efficacy of PD-1 inhibitors in advanced STS including nivolumab, pembrolizumab, and geptanolimab. The SARC028 study including 40 patients with advanced STS treated with pembrolizumab showed that the ORR was 17.5%, the median PFS was 18 weeks, and the 12-week PFSR was 55% (26). The Alliance A091401 trial including 42 patients with advanced sarcoma treated with nivolumab monotherapy showed that the ORR and median PFS were 5% and 1.7 months, respectively (27). Although only a low percentage (12.8%) of ASPS patients were included in this study, the combination therapy showed promising outcomes with a PFSR of 48%, ORR of 32%, median PFS of 7.0 months, and median OS of 17.2 months. The efficacy may be attributed to the transformation of the microenvironment of soft tissue sarcoma from cold to inflamed by promoting infiltration of effector immune cells (eg, dendritic cells and T-cells) and hampering immunosuppressive cells (eg, myeloid-derived suppressive cells and regulatory T cells) in the tumor microenvironment (7, 12, 28). The change translates into benefits for long-term survival metrics such as 6-month PFS and OS. In this study, one patient with UPS who experienced lung metastasis treated with anlotinib with camrelizumab had stable disease for more than two years after the failure of apatinib monotherapy. The significant survival benefit may be mainly due to the synergistic effect of combination therapy. Several recent studies investigated the efficacy of PD-1 inhibitors versus PD-1 inhibitors combined with other antitumor therapies in advanced STS. The Alliance A091401 study including 76 patients with metastatic sarcomas showed that a better ORR could be observed in patients treated with nivolumab and ipilimumab compared with nivolumab (16% vs. 5%) (27).

Recent studies explored the potential efficacy improvement of immunotherapy plus antiangiogenic agents in advanced STS. Due to the rarity and heterogeneity of STS, a phase II study including 36 patients with advanced STS (12 with ASPS and 24 with non-ASPS) treated with axitinib plus pembrolizumab showed that the ORR of the whole population was 25% (8/32) (29), the median PFS was 4.7 months, the 6-month PFSR was 46.9%, and the median OS was 18.7 months. The 6-month PFSR in patients with non-ASPS treated with the combination treatment was slightly higher than that of historical axitinib monotherapy (38.1% vs. 25-30%), and more tumor response (CR and PR) in patients with ASPS treated with combination treatment were reported compared to in-patients treated with each agent monotherapy (54.5% vs. 35%). A phase II study including 68 patients (16 patients in phase Ib and 52 patients in phase II) with advanced STS receiving sunitinib and nivolumab met the key endpoint of 6-month PFSR (30). For patients in phase II, the 6-month PFSR was 48%, the ORR was 13%, the median PFS was 5.6 months, and the m-OS was 24 months. The histopathological subtypes in that study were consistent with the present study characterized by a low proportion of ASPS and a wide range of subtypes. Notably, the median overall survival was significantly longer for the entire population, which was in line with our study. A recent study enrolled 30 patients with locally advanced or metastatic STS (12 ASPS and 18 non-ASPS) treated with anlotinib plus penpulimab was reported (31). For the total population, the 6-month PFSR was 53.85%, the ORR was 36.67%, and the median PFS was 7.85 months. In patients with ASPS, the PFSR at six months was 100%, the ORR was 75%, and the median PFS was 23.06 months. The ORR for patients with non-ASPS was 11.11%, the median PFS was 2.89 months, and the median OS was 10.58 months. Interestingly, the median PFS of seven patients with synovial sarcoma in the study was 2.07 months but their OS was significantly prolonged, reaching 17.31 months. Therefore, we thought that the efficacy of antiangiogenic drugs combined with PD-1 inhibitors varied in histopathological subtypes. The combination therapy could not only improve ORR and PFS, but also potentially improve OS in patients with ASPS due to the typical molecular mismatch repair deficiency and aberrant upregulation of HIF1α and VEGF (32). However, the combination therapy may only have advantages in long-term survival (PFSR at six months and OS) rather than traditional short-time efficacy in patients with non-ASPS. That was consistent with the long tail effect of the immunomodulatory therapy and its impact on durable survival benefits from subsequent treatments in diverse malignancies. Therefore, the antitumor efficacy of immunotherapy may be underestimated by traditional short-term efficacy indicators including ORR, DCR, and 3-month PFSR.

Predictive criteria to identify patients who benefit from the combination treatment would be of high value in the routine clinical setting. To date, several approaches to identify prognostic biomarkers for the efficacy and response of multi-tyrosine kinase inhibitors and/or PD-1 inhibitors have met minimal successes in advanced STS (6). In this study, we preliminarily investigated the association between prognosis with easily available clinical indicators including baseline NLR, baseline LMR, and histopathological types. Similar to our study, Wilky et al (29) suggested that a high baseline NLR was related to PD in 36 patients with advanced STS treated with axitinib plus pembrolizumab. Sato et al (33) reported that a low baseline NLR could be a predictive biomarker for better durable clinical benefit and OS in patients with advanced STS treated with pazopanib. Brewster et al (34) suggested that a low absolute lymphocyte count at baseline was related to the lower probability of 5-year overall survival among 634 patients with localized bone and soft tissue sarcoma. The mechanism between peripheral blood inflammatory markers and prognosis has not been fully established, the potential mechanisms are that NLR not only reflects antitumor immune status due to the potential function of neutrophils and lymphocytes in tumor progression but also has a critical effect on the balance of angiogenesis, the immune system, and the cytokine profile (33). Recent studies have shown that a low peripheral blood lymphocyte count may limit the ability of complex autoregulation resulting in an unendurable antitumor response by anti-inflammatory molecules (eg. regulatory T cells and tumor-associated macrophages) and pro-inflammatory molecules (eg. tumor-infiltrating lymphocytes) (35).

Overall, the combination of antiangiogenic agents and PD-1 inhibitors showed tolerable toxicity. The most common was hypothyroidism (56.4%), followed by fatigue (46.2%) and hypertriglyceridemia (43.6%), and the AEs ≥ grade 3 were pneumothorax, hypertension, and cytokine release syndrome. Although the initial dose of apatinib in advanced gastric adenocarcinoma and gastroesophageal junction cancer is recommended at 850mg/day, the starting dose of apatinib in this study was 500mg/day, which is similar to the previous reports of advanced soft tissue sarcomas. Only 25% (2/8) of patients treated with camrelizumab combined with anti-angiogenesis suffered capillary hemangiomas, the rate was lower than that of camrelizumab monotherapy in cancers, which may be related to the inhibition of angiogenesis. A high proportion of AEs ≥ grade 3 (2/8) were observed in patients treated with the combination therapy of apatinib and camrelizumab. This suggests that the patients should be followed up closely and the dose of agents should be reduced in time. Grade 4 cytokine release syndrome was observed in one patient with synovial sarcoma two weeks after the first cycle combination therapy of apatinib and camrelizumab, which has rarely been reported in targeted therapy and/or immunotherapy.

Due to the convenience and potential effectiveness, numerous clinical studies have been conducted on this combination therapy strategy for malignant tumors, some of which yielded outcomes. We retrieved the clinical trial website (https://clinicaltrials.gov) and found that there are three clinical studies in the pipeline for antiangiogenic agents and PD-1 inhibitors aside from the published clinical studies in advanced STS. The first one is a phase 2 study which plans to enroll five groups of patients with advanced sarcoma who would be treated with lenvatinib plus pembrolizumab (NCT04784247) (36). The histopathological type includes LMS, high-grade UPS, vascular sarcomas, osteosarcoma, and other STS, and the primary study endpoint is ORR. The second one is a phase 2 study in which patients with advanced STS, Ewing’s sarcoma, osteosarcoma, and UPS would receive pembrolizumab plus cabozantinib and the primary endpoint is the efficacy of the combination therapy (NCT05182164) (37). The last one is a phase 2 study of osteosarcoma patients treated with regorafenib and nivolumab with a primary endpoint of the difference in 4-month progression-free survival from historical controls (NCT04803877) (38). These suggest that the exploration of the efficacy of this treatment regimen in different histological types or in specific candidate populations may be a trend for future research. Additionally, there are several studies to screen candidates who could benefit from immunotherapy and/or targeted therapy and investigate the tumor microenvironment and biomarkers of the efficacy of targeted and/or immunotherapy in patients with sarcoma, such as BIOVAS (NCT04072042) (39) and HIFU-UPS (NCT04123535) (40). We are designing a company-sponsored randomized controlled phase 2 clinical trial that aims to evaluate the efficacy of the combination therapy versus targeted therapy alone and explore prognostic biomarkers in patients with advanced ASPS.

This study has some limitations. First, due to the characteristics of a retrospective study, heterogeneity of prior treatment and histopathological types, and the small sample size of each type, the promising efficacy of the combined therapy still needs to be confirmed by large sample prospective studies in the future. However, this does not diminish the value of this study as a reference for future research. Second, this study preliminarily analyzed the correlation between clinical characteristics and prognosis. Regrettably, these characters did not include molecular analyses and immunological profiles such as PD-L1, VEGFR, PDGFR, tumor mutation burden, and microsatellite instability, which may give us more insight into the relationship between the efficacy of combination therapies and molecular mechanisms. Finally, although the median follow-up time in this study was nearly 1.5 years, which may be sufficient for patients with advanced non-ASPS, further follow-up was needed for patients with good prognoses, including ASPS, extraosseous chondrosarcoma, and LMS.

## Conclusion

Antiangiogenic agents plus PD-1 inhibitors showed promising activity and favorable toxicity in patients with advanced STS, especially patients with ASPS. A low baseline NLR might serve as a reliable biomarker for 6-month PFSR, ORR, and PFS. A randomized, controlled study of antiangiogenesis-immunotherapy versus antiangiogenesis as second-line treatment is ongoing.

## Data availability statement

The original contributions presented in the study are included in the article/**Supplementary Material**. Further inquiries can be directed to the corresponding author.

## Ethics statement

Approval of this study was achieved from the Institutional Review Board of each institute according to the declaration of Helsinki. Written informed consent for treatment from each patient was obtained before either regimen.

## Author contributions

All authors listed have made a substantial, direct, and intellectual contribution to the work and approved it for publication.
