# Supplementary material for: The efficacies and biomarker investigations of antiangiogenic agents and PD-1 inhibitors for metastatic soft tissue sarcoma: A multicenter retrospective study
Source: Front Oncol. 2023 Feb 22;13:1124517. doi: 10.3389/fonc.2023.1124517 (PMC9992731; doi:10.3389/fonc.2023.1124517)
Supplement: Supplementary file 1 [file Table_1.docx]

**Table S1**. Univariate analyses of factors associated with progression-free survival and overall survival

| Characteristic | Category |  | PFS | |  | OS | |
| --- | --- | --- | --- | --- | --- | --- | --- |
|  |  |  | Univariate | |  | Univariate | |
|  |  |  | HR (95%CI) | *p*-Value |  | HR (95%CI) | *p*-Value |
| Age | ＜40 vs. ≥40Years |  | 0.53(0.21-1.32) | 0.17 |  | 0.43(0.14-1.26) | 0.12 |
| Sex | Male vs. female |  | 0.71(0.31-1.66) | 0.43 |  | 0.78(0.27-2.26) | 0.65 |
| ECOG | 0 vs.≥1 |  | 0.82(0.32-2.13) | 0.69 |  | 0.91(0.31-2.66) | 0.85 |
| Histology | ASPS vs. non-ASPS |  | 0.4(0.06-2.57) | 0.33 |  | 0.77(0.06-9.33) | 0.84 |
| Metastatic organs | Single vs multiple organs |  | 0.68(0.27-1.71) | 0.42 |  | 0.71(0.24-2.06) | 0.53 |
| Primary lesions | Extremity vs. other |  | 1.21(0.46-3.15) | 0.71 |  | 1.12(0.34-3.63) | 0.84 |
| Previous PD-1 inhibitor | ≥1 vs. 0 |  | 1.79(0.33-9.62) | 0.5 |  | 2.14(0.26-16.6) | 0.46 |
| Previous tyrosine-kinase inhibitor | ≥1 vs. 0 |  | 1.44(0.41-4.99) | 0.57 |  | 1.47(0.32-7.05) | 0.62 |
| No. of previous chemotherapies | 0–1 vs. ≥2 |  | 0.84(0.32-2.15) | 0.72 |  | 1.22(0.34-4.36) | 0.75 |
| LMR | ≥2.8 vs.＜2.8 |  | 0.62(0.24-1.59) | 0.32 |  | 0.35(0.09-1.33) | 0.12 |
| NLR | ≥4.0 vs.＜4.0 |  | 4.28(1.47-12.41) | 0.01 |  | 4.77(1.48-15.40) | 0.01 |
| ALC | ＜1000 vs.≥1000/µL |  | 2.78(1.13-6.81) | 0.03 |  | 1.54(0.48-4.89) | 0.45 |

Abbreviations: ALC, absolute lymphocyte count; CI, confidence interval; ECOG PS, Eastern Cooperative Oncology Group performance status; HR, hazard ratio; NLR, neutrophil-to-lymphocyte ratio; PD-1inhibtor, programmed death-1 inhibitor; LMR, lymphocyte to monocyte ratio; PFS, progression-free survival; OS, overall survival.
