# Supplementary figures and images for: The efficacies and biomarker investigations of antiangiogenic agents and PD-1 inhibitors for metastatic soft tissue sarcoma: A multicenter retrospective study
Source: Front Oncol. 2023 Feb 22;13:1124517. doi: 10.3389/fonc.2023.1124517 (PMC9992731; doi:10.3389/fonc.2023.1124517)

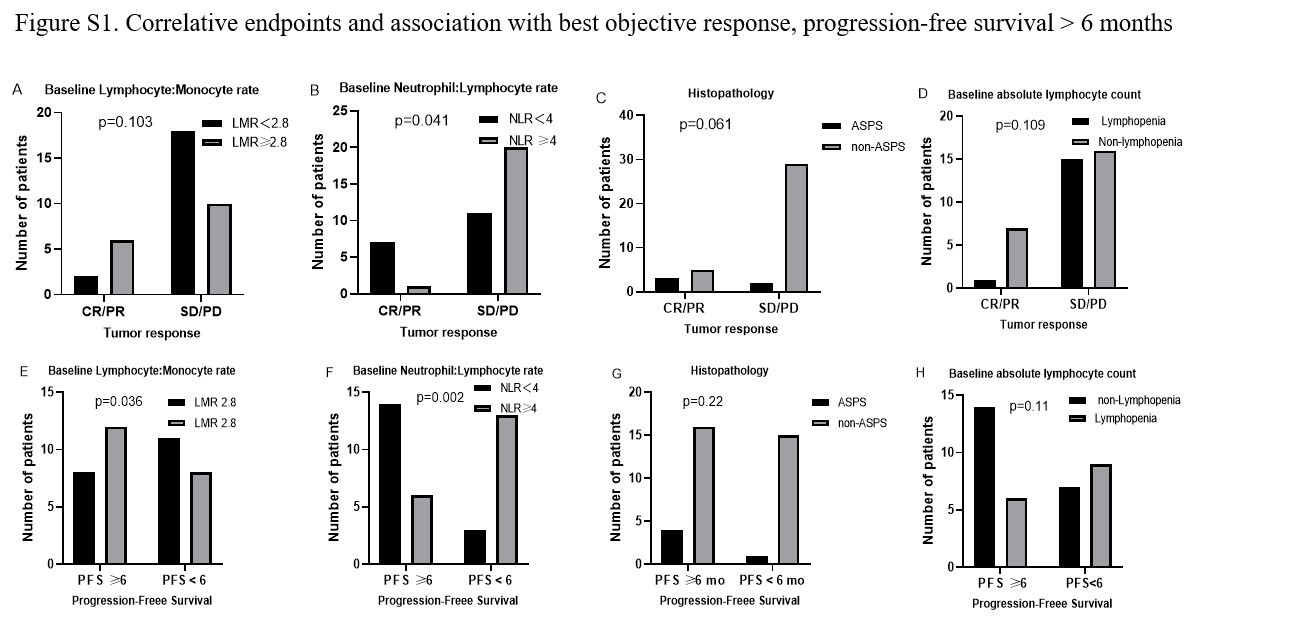

Supplement: Supplementary file 2 [file Image_1.jpeg]
